# Variation in the Accumulation of Phytochemicals and Their Bioactive Properties among the Aerial Parts of Cauliflower

**DOI:** 10.3390/antiox10101597

**Published:** 2021-10-12

**Authors:** Natalia Drabińska, Maja Jeż, Mariana Nogueira

**Affiliations:** 1 Department of Chemistry and Biodynamics of Food, Institute of Animal Reproduction and Food Research of Polish Academy of Sciences, 10-748 Olsztyn, Poland; msnogueira@ucp.pt; 2 Food Volatilomics and Sensomics Group, Faculty of Food Science and Nutrition, Poznan University of Life Sciences, 60-637 Poznań, Poland; 3 Department of Chemical and Physical Properties of Food, Institute of Animal Reproduction and Food Research of Polish Academy of Sciences, 10-748 Olsztyn, Poland; m.jez@pan.olsztyn.pl; 4 Faculty of Biotechnology, Universidade Católica Portuguesa, 4169005 Porto, Portugal

**Keywords:** cauliflower, leaves, florets, stem, glucosinolate distribution, antioxidant activity, phytochemicals

## Abstract

Vegetables from the Brassicaceae family are excellent sources of bioactive phytochemicals and may reduce the risk of chronic diseases. Variation of phytochemicals in the edible part of cauliflower is known. However, information about the distribution of bioactive and nutritive compounds as well as antioxidant activity among aerial organs of cauliflower is unavailable. Therefore, this study aimed to evaluate the distribution of glucosinolates (GLS), phenolics, flavonoids, chlorophylls, nutritive compounds and antioxidant capacity between the aerial parts of the common variety of cauliflower and to evaluate whether these changes contribute to the differences in the antioxidant capacity between the plant organs. Our study showed that all the aerial organs of cauliflower are a rich source of health-promoting bioactive compounds, including GLS, phenolics and flavonoids, exhibiting antioxidant capacity. The highest contents of phytochemicals and the highest antioxidant capacity were found in leaves. Cauliflower organs were also found to be rich in nutritive compounds, including minerals, proteins and amino acids. Our study showed that the non-edible organs, such as stems and leaves, being neglected parts of cauliflower, if not consumed as the main ingredient, can be used as additives for developing new, functional foodstuff.

## 1. Introduction

Cauliflower (*Brassica oleracea* L. var. *botrytis*) is a commonly consumed vegetable belonging to the Brassicaceae family, having a substantial content of dietary fiber, vitamins, minerals and bioactive compounds [1]. The characteristic flavor of cauliflower, typical for other cruciferous vegetables, is related to the presence of sulfur compounds and their derivatives. These sulfur compounds are named glucosinolates (GLS) which are plant defensive metabolites found in all other *Brassica* plants. Depending on the side chain of GLS, three groups can be distinguished, namely aliphatic, indolic and aromatic GLS, which are derived from different amino acid precursors [2]. During the disruption of plant tissue, GLS stored in the vacuoles mix with the enzyme myrosinase, which hydrolyzes GLS to several breakdown products, depending on the side chain of GLS and the presence of specific proteins [3]. Two main groups of breakdown products of GLS are isothiocyanates and nitriles, which are toxic and noxious for herbivores. On the other hand, epidemiological studies suggested that the higher consumption of vegetables belonging to the *Brassica* genus can reduce the risk of cancerogenesis in humans, mainly due to the presence of GLS breakdown products [4]. Isothiocyanates, the same compounds important in the plant defense system, have been proven to be potent inducers of phase II detoxification enzymes and have anti-inflammatory properties [5,6]. Apart from GLS, the edible part of the cauliflower contains phenolic acids, flavonoids, carotenoids and ascorbic acid [1]. It is suggested that these phytochemicals exert health-promoting properties, mainly due to the antioxidant capacity, which may protect the human body against damage caused by reactive oxygen species [7,8].

The consumption of cauliflower is mainly limited to flowers, which is the main, white part. However, the by-products, like stem and leaves, could also be a valuable source of bioactive compounds. By-products of other *Brassica* vegetables, such as broccoli, were found to be a good source of bioactive compounds, which has gained some scientific attention [9]. Broccoli leaves were found to have a rich profile of phytochemicals, very similar to the edible part, and thus have been used as an attractive additive to new functional food [10]. Similar attempts have been conducted with cauliflower [11,12]; however, the potential of cauliflower by-products has not been fully characterized.

The profile and content of phytochemicals in plants are usually influenced by plant variety, organ, developmental stage, drought stress, insect feeding and other genotypic and environmental factors [13,14,15,16,17]. Variation of GLS and other bioactive compounds in the edible part of cauliflower has been previously described [1,18,19]. However, the information about the distribution of phytochemicals, nutritive compounds and antioxidant activity among aerial organs of cauliflower is unavailable. Therefore, the objective of this study was to evaluate the distribution of GLS, phenolics, flavonoids, chlorophylls, nutritive compounds and antioxidant capacity between the aerial parts of the common variety of cauliflower and to evaluate whether these changes contribute to the differences in the antioxidant capacity between the plant organs. The results presented in this work will reveal the nutraceutical potential of different cauliflower aerial organs and provide consumer information on the possible potential of, so far, non-edible organs.

## 2. Materials and Methods

### 2.1. Plant Material

Three cauliflowers (*Brassica oleracea* Botrytis group) of Guideline F1 cultivar containing the leaves were purchased in a local market in Olsztyn, Poland. Cauliflowers were washed in tap water to remove soil residues and separated into florets, leaves and stems. Only the mature leaves without any sign of mechanical damage and disease were selected. Leaves, florets and stems were blanched in boiling water for 1, 2 and 2 min, respectively, to inactivate enzymes responsible for hydrolyzing the biologically active compounds. From leaves, the petioles and the main midribs were removed. Stems and florets were cut in cubes of approximately 3 cm. All the samples were freeze-dried and ground into a fine powder with particle size <0.60 mm. Powders were kept in the freezer (−24 °C) until analysis.

### 2.2. Color Analysis

The color of freeze-dried cauliflower organs was evaluated using a HunterLab ColorFlex (Hunter Associates Laboratory, Inc, Virginia, USA). The measurements were performed through a 3-cm-diameter diaphragm containing an optical glass. Powders were placed in the clean glass container to cover the bottom surface and were covered from the light with a black lid. The color was expressed in accordance with CIELab system and the parameters determined were lightness: L* = 0 (black)–100 (white); and chromatic components: a* = −a* (greenness)–+a* (redness), and b* = −b* (blueness)–+b* (yellowness). Values were the mean of at least nine replicates.

### 2.3. Proximate Composition

The proximate composition was determined using standard methods in freeze-dried samples [20]. Mineral content (ash) was determined using the gravimetric method by burning in a muffle furnace for 1 h at 585 °C (AOAC 923.03). The protein content was analyzed using the Kjeldahl method (*N* × 6.25 for nitrogen to protein conversion) (AOAC 979.09). Finally, the fat content was determined using Soxhlet extraction with hexane (AOAC 923.03). The total carbohydrate content was estimated by subtracting the protein, fat and ash content from 100%.

### 2.4. The Analysis of Glucosinolates

The GLS content was analyzed using a method described by Drabińska et al. [10], following the method of the Official Journal of the European Communities [21]. Briefly, approximately 0.2 g of the dried sample was extracted with 3 mL of 70% boiling methanol by homogenization. The extraction was repeated three times. Supernatants were collected in a 10 mL volumetric flask and filled to the line. Next, desulphation was performed using *Helix pomatia* sulphatase. Desulpho-GLS were analyzed using a high-performance liquid chromatography (HPLC) system with an autosampler (LC-20) and an SPD-M20 DAD detector (Shimadzu, Japan). The separation was performed using a LiChrospher^®^ 100 RP-18 (5 μm, 250 × 4 mm) column (Merck, Darmstadt, Germany) with a flow rate of 1.2 mL/min in a gradient of water (phase A) and 20% acetonitrile (phase B). Glucosinolates were identified and quantified in response to the commercial standards purchased from PhytoPlan^®^ (Heidelberg, Germany).

### 2.5. The Analysis of Amino Acids

A 0.1 g quantity of freeze-dried sample was extracted with 3 mL of methanol solution (50% *v*/*v*) by shaking at 500 rpm for 20 min at 50 °C using a thermomixer (Thermomixer, Eppendorf, Poland). The supernatant obtained after centrifugation (10,000 rpm for 15 min) was directly analyzed using the EZ:faast™ kit for free (physiological) amino acids (Phenomenex, Aschaffenburg, Germany) according to the producer’s recommendations. The analytical procedure involves solid-phase extraction of 100 μL of extract, followed by derivatization and liquid–liquid extraction. Amino acids were separated in the Agilent 7890A gas chromatograph coupled with the 5975C mass selective detector, 7683B auto-injector (Agilent Technologies, Santa Clara, CA, USA) and a data station containing the NIST/EPA/NIH Mass Spectral Library (Version 2). The compounds were separated in a ZB-AAA EZ:faast™ capillary column (10 m × 0.25 mm). The carrier gas was helium (1.5 mL/min). The samples (2 µL) were injected in split mode (1:15). The oven temperature was initially set at 110 °C and then increased to 320 °C (30 °C/min). Injector and ion source temperatures were 250 °C and 240 °C, respectively. Mass spectra were obtained by electron ionization (EI) over the range of 35–550 m/e. Electronic impact energy was 70 eV. Amino acids were identified using calibration standards for each amino acid, and quantitative analysis was performed relative to the internal standard (norvaline). The limit of detection was 1 nmol/mL, and the precision of quantification was below 15% for all amino acids, as stated by the manufacturer. 

### 2.6. Extract Preparation

A total of 150 mg of the freeze-dried sample was extracted with 1 mL of methanol–water solution (80:20; *v*/*v*). Ultrasonic vibration (30 s) and vortexing (30 s) were repeated three times, and the samples were centrifuged for 10 min at 13,000 rpm at 4 °C. The above step was repeated five times, and the supernatants were collected into a 5 mL measuring flask. Methanol extracts were prepared in triplicate.

### 2.7. Total Phenolic Content

Total phenolic content (TPC) was determined using the method described by Drabińska et al. [10]. Briefly, 15 μL of methanol extracts and 250 μL of the Folin–Ciocalteu reagent (diluted with water 1:15, *v*/*v*) were placed in microplate wells (350 µL; PS, Porvair, Bioanalytic, Poland), and the mixture was incubated in the dark for 10 min at ambient temperature. Then, 25 μL of 20% sodium carbonate was added to each well, and the mixture was incubated for another 20 min. The microplate was shaken automatically before readings, and absorbance was measured at λ = 755 nm with the Infinite M1000 PRO plate reader (Tecan Group AG, Mannedorf, Switzerland). The calibration curve was obtained with gallic acid (GAE) in the concentration range of 0.062 to 0.500 mg/mL (R^2^ = 0.999), and the results were expressed in mg of gallic acid equivalents (GAE) per one gram of dry matter (g DM) of cauliflower aerial part.

### 2.8. Total Flavonoid Content

The total flavonoid content (TFC) was determined using the method described by Horszwald and Andlauer [22]. A total of 25 µL of the sample was mixed with 75 µL of an aqueous solution of ethanol (95:5; *v*/*v*) in the microplate well. Next, 5 µL of 10% AlCl_3_·6H_2_O, 5 µL of 1 M potassium acetate and 140 µL of deionized water were added, mixed and incubated at ambient temperature. After 30 min, the absorbance was measured at λ = 415 nm against deionized water with an Infinite M1000 PRO plate reader (Tecan Group AG, Mannedorf, Switzerland). The total flavonoid content was determined using a standard curve with rutin at a concentration range of 0.003–0.332 mg/L (R^2^ = 0.999). Total flavonoid content was expressed as mg of rutin equivalents (RUT) per one gram of dry matter (g DM) of cauliflower aerial part.

### 2.9. Ferric Reducing Antioxidant Potential

The ferric reducing antioxidant potential (FRAP) assay was performed according to the method proposed by Horszwald and Andlauer [22] and described by Drabińska et al. [10]. Briefly, 50 μL of the methanol extract and 275 μL of a freshly prepared FRAP reagent (5 mL of 10 mM 2,4,6-tri(2-pyridyl)-s-triazine in 40 mM hydrochloric acid combined with 5 mL of 20 mM ferric (III) chloride solution and 50 mL of 0.3 mM acetate buffer, pH 3.6) was placed in microplate wells. The mixture was incubated for 5 min at ambient temperature, and the absorbance at λ = 593 nm was measured in a microplate reader (Infinite M1000 PRO plate reader, Tecan Group AG, Mannedorf, Switzerland). 6-Hydroxy-2,5,7,8-tetramethylchroman-2-carboxylic acid (Trolox) was used for calibration in the concentration range of 0.30–250 μmol/L (R^2^ = 0.997), and the results were expressed in μmol TE (Trolox equivalents)/g DM of cauliflower aerial part.

### 2.10. Trolox Equivalent Antioxidant Capacity Assay with ABTS

The Trolox equivalent antioxidant capacity (TEAC) assay was performed based on the method described by Horszwald and Andlauer [22] and Drabińska et al. [10] using a 2,2′-azinobis-(3-ethylbenzothiazoline-6-sulphonic acid) diammonium salt (ABTS solution. Briefly, ABTS solution was diluted with methanol to the absorbance level of 0.70 ± 0.02 at 734 nm. Then 10 μL of methanol extract was placed in microplate wells. Then, 270 μL of the ABTS solution was added, and the reaction was carried out at 30 °C in the dark for 6 min, and the absorbance was measured at 734 nm with a microplate reader (Infinite M1000 PRO plate reader, Tecan Group AG, Mannedorf, Switzerland). Trolox was used for standard calibration at a concentration range of 0.200−0.800 µmol/L (R^2^ = 0.993), and the results were expressed in µmol TE/g DM of cauliflower aerial part.

### 2.11. The Analysis of Chlorophylls

The content of chlorophylls was analyzed using the method described by Lichtenthaler [23]. Briefly, 400 mg of sample was mixed with 5 mL of an aqueous solution of acetone (80:20; *v*/*v*) and centrifuged for 15 min at 14,000 rpm. The absorbance was measured in the supernatants at 663 nm and 647 nm for chlorophyll a and b, respectively. The results were expressed as µg per gram of the dry matter (µg/g DM).

### 2.12. Statistical Analysis

All analytical measurements were performed in triplicate for three individual cauliflowers, giving in total nine replicates for each aerial part. The results were analyzed using a one-way analysis of variance (ANOVA). The significance of differences between the samples was determined by Fisher‘s LSD test at *p*-value < 0.05. Correlations between parameters were analyzed using a Pearson correlation coefficient test. All statistical analyses were performed using GraphPad Prism version 8.0.0 for Windows (San Diego, CA, USA) software.

## 3. Results

The results regarding the instrumental color analysis of aerial parts of cauliflower are presented in Table 1. The color of florets and stems seemed to be similarly creamy; however, statistically significant differences were observed between these samples for all the color parameters. Not surprisingly, the most distinguishing was the color of leaves, which was characterized by higher greenness (negative a* value), lower lightness (L* value) and higher yellowness (positive b* value) compared to the other organs.

The distribution of GLS between the aerial parts of cauliflower is presented in Table 2. In total, six identified GLS and two unidentified GLS were determined in all cauliflower organs. The highest content of GLS was determined in cauliflower leaves, mainly due to the indole GLS. Irrespectively on the organ, the proportion of indole GLS was much higher than the aliphatic ones, considering that the UV spectra of the two unidentified GLS fit more to the aliphatic GLS. Aliphatic GLS, including progoitrin and sinigrin, were determined in the highest concentrations in stems. Regarding the distribution of the individual compounds, the dominant GLS in florets was glucobrassicin, while in leaves and stems, the major GLS was neoglucobrassicin.

**Figure 1:**
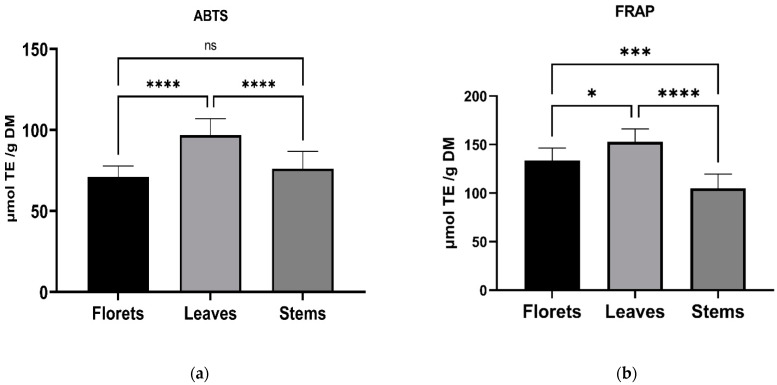
The antioxidant capacity of the aerial parts of cauliflower, expressed the mean ± SD (*n* = 9) analyzed using (**a**) ABTS and (**b**) FRAP assays. ABTS—2,2′-azino-bis (3-ethylbenzothiazoline-6-sulfonic acid) (ABTS)—radical cation-based assay; FRAP—ferric reducing antioxidant potential (FRAP) assay; TE—Trolox equivalents. (ns) – not significant; (*)—*p*-value < 0.05; (***)—*p*-value < 0.001; (****)—*p*-value < 0.0001.

**Figure 2:**
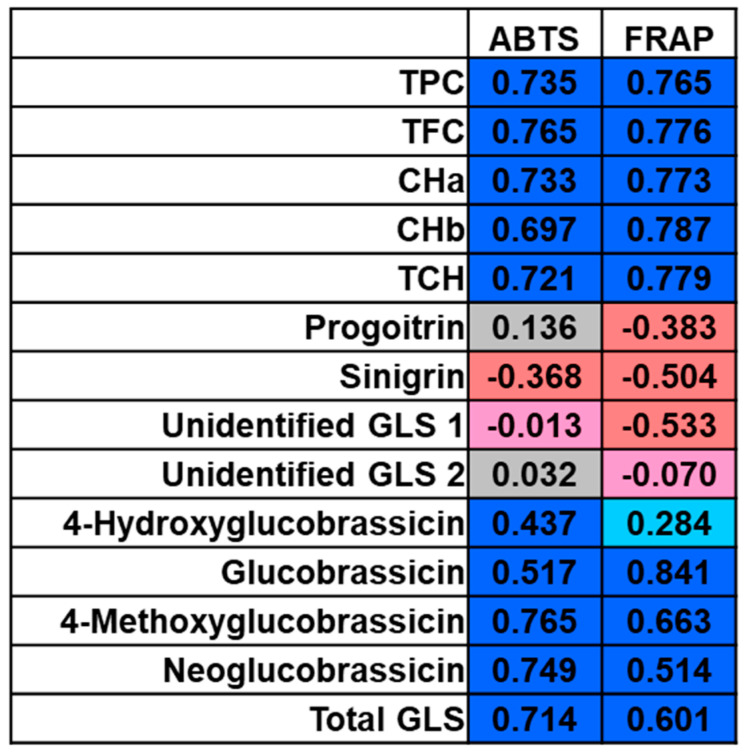
Heatmap representation of the correlations between antioxidant capacity and the phytochemicals analyzed in the aerial parts of cauliflower. ABTS—2,2′-azino-bis (3-ethylbenzothiazoline-6-sulfonic acid) (ABTS)—radical cation-based assay; FRAP—ferric reducing antioxidant potential (FRAP) assay; TPC–total phenolic content; TFC—total flavonoid content; CHa—chlorophyll a; CHb—chlorophyll b; TCH—total chlorophylls; GLS—glucosinolate.

The total content of phenolic compounds, flavonoids and chlorophylls in the aerial parts of cauliflower is presented in Table 3. Not surprisingly, the highest content of chlorophylls was determined in leaves, which were the greenest. The chlorophyll content did not vary between florets and stems. The TPC and TFC were the highest in leaves, while in stems and florets, these values were similar. This observation found confirmation in the antioxidant capacity measured in two different assays (Figure 1). In both assays, leaves showed the highest antioxidant activity. However, the FRAP assay showed also the difference between the antioxidant potential of florets and stems, while in the ABTS assay, these values were similar. The high positive correlations were found between the antioxidant capacity and TPC, TFC and TCH (Figure 2).

The proximate composition, including mineral content, protein, fat and carbohydrates, is presented in Table 4. Cauliflower was found to be a rich source of minerals. The highest ash content was determined in stems, while the mineral content was similar in florets and leaves. Moreover, cauliflower was characterized by a high protein abundance, with florets being the most reached source. Similarly, the highest content of carbohydrates was noted in florets; however, this value did not differ significantly between the aerial parts of cauliflower. Similar fat content was determined for leaves and stems, which was two times higher than in florets.

The profile and content of amino acids in the aerial parts of cauliflower are presented in Table 5. In total, 17 amino acids were determined in cauliflower in all the analyzed organs. The highest content of amino acids was noted for leaves, which was higher by 49 and 83% for stems and florets, respectively. In all the aerial parts, the dominant amino acid was glutamine; however, the distribution of this amino acid between the organs varied significantly. The highest abundance of glutamine was noted in leaves, which was 2-fold and 4-fold higher than in stems and florets, respectively. The glutamine content contributed mainly to the total content of non-essential amino acids, being the highest in leaves. However, the opposite phenomenon was observed for the essential amino acids. The total content of essential amino acids was the highest in florets and the lowest in leaves.

## 4. Discussion

Our study showed that the distribution of the phytochemicals and the nutritional quality differ significantly between the aerial parts of cauliflower. The results demonstrated that all organs of cauliflowers are a rich source of GLS, antioxidants and amino acids, but their levels in individual organs varied considerably. These results suggest that, in addition to the edible part, other organs of cauliflower, especially the leaves, can be a valuable food product or could potentially be used to produce new functional foods.

Although the distribution of the defense compounds in plants has gained much scientific attention [24,25,26], most studies have focused on the individual group of compounds, and only a small number of studies have exploited changes in the phytochemicals in cauliflower [18]. In this study, we showed the distribution of the defensive and nutritive chemicals and the antioxidant capacity between the aerial parts of cauliflower, which contribute to the survival of plants and have high importance for the consumers.

In 1974, McKey developed optimal defense theory (ODT) to explain the distribution of defensive chemicals within a plant [27]. According to this concept, the distribution of phytochemicals in the plant organs depends on their value, defined as a significance in primary functions such as growth and reproduction. ODT predicted that flowers, fruits and younger leaves are the most vulnerable and the most important to the plant [28]. In our study, the highest content of the defense chemicals (GLS, phenolics, flavonoids) was detected in leaves, which suggests that leaves would be the most important organ for cauliflower according to the ODT. Flowers seems to be more critical for reproduction than leaves, thus our study does not confirm the concept suggested by ODT. Our results are partly in line with the meta-analysis conducted to check the predictive utility of ODT, showing no much difference in the distribution of defense compounds between leaves and flowers in the majority of plants, showing that the ODT has to be considered carefully [28]. The authors explained that not every flower will set the fruit, and thus flowers are less valuable than the more photosynthetically active leaf, which could produce enough energy to mature several fruits.

Our study contradicts another concept developed by Tsunoda et al. [26], who suggested that most core organs would have higher concentrations of defensive compounds than more distal parts. According to their theory, the stem is the most important organ for the plant since it houses the main transportation and signal communication system. The damage of the stem would result in the loss of integrity, leading to the loss of also the undamaged, distal parts [26]. In our study, the highest content of all GLS was found in leaves, not as suggested in the stem. However, despite the total GLS content, the highest abundance of aliphatic GLS was observed in stems, which is partly in line with the theory of Tsunoda et al. [26]. Aliphatic GLS are considered more toxic and thus are distributed to the first-order organ. According to Brown et al. [29], indole GLS is the dominant group in the more mature leaves. The authors reported that the total GLS content increases with the age of leaves, which can explain our finding, since fully mature leaves were analyzed in our study.

The possible explanation of the distribution of defense compounds to leaves in cauliflower observed in our study can be related to the morphological structure. In cauliflower, leaves are the outermost organ, protecting other aerial parts, both flowers and stems. Hence leaves are more exposed to be attacked by herbivores and require higher phytochemical protection than other organs. Several previous studies showed that both transcription factor-related and GLS biosynthesis genes show differences in expression in different plant organs such as seeds, stems, leaves and flowers, which differ between plants even belonging to the same subspecies [18,30]. In the study on Chinese cabbage [30], the highest content of GLS in aerial parts was noted for flowers, followed by young leaves and stems. However, the flowers of Chinese kale are fully exposed during flowering, contrary to cauliflower.

Antioxidants comprise a broad and heterogeneous group of compounds that share the common task of stopping, retarding or preventing the oxidation of an oxidizable substrate [31]. Phenolic compounds, flavonoids, vitamin C and chlorophylls, which exhibit high antioxidant activity, were suggested to decrease the risk of developing many non-communicable diseases and several types of cancer [32,33,34]. Antioxidants can act directly, such as catalase, metal chelators and flavonoids, or indirectly, stimulating the efficacy of the physiological antioxidant defenses such as GLS and their breakdown products [31]. The synergistic effect of multiple antioxidants reflects the plant antioxidant capacity. Our study showed high positive correlations between the main antioxidants (TPC, TFC and chlorophylls), GLS and the antioxidant activity (Figure 2), implying that these antioxidants mainly contributed to the total antioxidant capacity. The contribution of similar compounds to the antioxidant capacity in *Brassica* vegetables was also suggested by other authors [35].

In our study, the highest antioxidant capacity was determined for leaves, irrespectively of the applied assay, which was in line with the highest contents of all bioactive compounds analyzed in this study. Similarly, in the study comparing antioxidant capacity between leaves and florets of different varieties of cauliflowers, in the white cauliflower varieties, the leaves were characterized by higher antioxidant capacity than florets [36]. Additionally, in the study by Kim et al. [37], who compared the antioxidant capacity of different organs of rocket salad, the greatest activity was found for the leaves, despite the lowest content of GLS. The high antioxidant potential of leaves and a big difference between this organ and flowers and stems can be related to the presence of defensive compounds and chlorophylls, which were detected in leaves in high amounts. In contrast, in flowers and stems, only a low quantity of chlorophylls was noted. Chlorophylls are the main naturally-occurring pigment characteristic of the photosynthetic organs that give the tissue a green color. Apart from their function in photosynthesis, studies showed that different pigments belonging to chlorophylls exhibit antioxidant activity [33]. Considering the high abundance of chlorophylls, especially chlorophyll a, in cauliflower leaves, which also corresponded to the color of analyzed samples, it can be assumed that chlorophylls contribute significantly to the overall antioxidant capacity.

Changes in the allocation of the nutritional resources during growth and modified fertilization, and the differences between the varieties have been analyzed repeatedly [38,39]. However, little is known about the distribution of the nutritive compounds between the different organs of *Brassica* vegetables, including cauliflower. During plant development, the distribution of nutritional quality expressed by the nitrogen amount has been reported to be opposite to the defensive compounds [24]. In our study, the highest content of protein and the lowest content of GLS was noted in florets, confirming this theory. Hong [40] reported that the nitrogen storage is the highest in the leaf-stem at the curd initiation, and then the translocation of water and nitrogen to the floret is observed. At harvest, the nitrogen concentration is higher in florets than leaf-stem. In our study, the highest protein content was observed in florets, but the difference between florets and leaf-stem was not as big as suggested by Hong [40]. However, the decrease in nitrogen was reported with the increase of the organ size [39,40]. Thus, the lower protein concentration in florets can be related to its dilution in the vegetable tissue at the harvest stage.

Plants are a rich source of amino acids, and their abundance in vegetables is of great significance, especially in terms of food. Amino acids are essential molecules for the proper functioning of the human body. The disturbance in the amino acid metabolism has been associated with several diseases and health issues such as cancer, autism spectrum disorders, severe pain and celiac disease [41,42,43,44]. Many of these diseases require adherence to elimination diets, in which the intake of individual amino acids can be disturbed. Therefore it is crucial to provide the amino acids with the diet, especially the essential ones. Amino acids constitute the most abundant chemical form in which nitrogen is transported in plants [45]. Amino acids play a pivotal role in physiological processes occurring in plants, such as growth, storage of nutrients and transportation [46].

The results of our study showed that cauliflower is a good source of free amino acids. Interestingly, the stem and leaves of cauliflower, which are considered waste, are also rich in amino acids. The cauliflower leaves have been found to have the highest content of amino acids from all the analyzed aerial parts. It can be related to the higher assimilation of nitrogen in photosynthetic leaf tissues [47]. Interestingly, the concentration of individual amino acids in all the aerial parts varied significantly, reflecting various functional roles of the individual amino acids in plants. Glutamic acid, glutamine, alanine and asparagine are primary products of nitrogen assimilation, and therefore their concentrations in leaves are higher [47,48]. On the other hand, lysine, proline and valine are derived mainly from protein degradation, and their concentrations increase during stress [48,49]. Amino acids are also precursors to the synthesis of defense chemicals. Tryptophan is the precursor of the indole GLS [2]. Therefore, its reduced quantities in leaves can be associated with the high production of glucobrassicin and its derivatives.

The results presented in this study can contribute to the utilization of cauliflower by-products as an additive to the food products, increasing the nutritional quality and nutraceutical potential of the foodstuff. The utilization of the by-products of fruits and vegetables has gained much scientific attention in recent years [50,51,52]. Broccoli leaves were found to be a good ingredient for sponge-cakes [2,13] and bread [14], increasing the content of bioactive compounds and antioxidant capacity without compromising the technological quality and sensory properties. Some attempts have been made for cauliflower leaves, showing that leaves rich in flavonoids and phenolic acids can be used to extend the shelf-life of meat products [11]. Moreover, cauliflower leaves have been incorporated into the malted wheat biscuits [12] and noodles [53], improving their nutritional potential without sacrificing the sensorial quality.

## 5. Conclusions

In conclusion, our study showed that all the aerial organs of cauliflower are rich sources of health-promoting bioactive compounds, including GLS, phenolics and flavonoids, exhibiting antioxidant capacity. The highest contents of phytochemicals and the highest antioxidant capacity were found in leaves, which to date are considered a by-product of cauliflower processing. Moreover, cauliflower organs have been found to be rich in nutritive compounds, including minerals, proteins and amino acids. The consumption of non-edible organs, such as stem and leaves, can be beneficial for human health due to the presence of bioactive compounds, and these aerial parts can be successfully used as ingredients for developing new, functional food products.

## Figures and Tables

**Table 1:** The color of the freeze-dried samples of the aerial parts of cauliflower expressed as the mean ± SD (*n* = 9).

	Florets	Leaves	Stems
L*	43.41 ± 0.00 ^b^	34.24 ± 0.01 ^c^	43.51 ± 0.00 ^a^
A*	0.23 ± 0.01 ^a^	−5.13 ± 0.02 ^c^	0.19 ± 0.01 ^b^
B*	6.49 ± 0.01 ^b^	13.36 ± 0.01 ^a^	5.66 ± 0.02 ^c^

Different letters in a row indicate statistically significant (*p*-value < 0.05) differences between the samples.

**Table 2:** The content of individual glucosinolates in the aerial parts of cauliflower expressed as mean ± SD (*n* = 9) in mmol/g DM. The total content of glucosinolates is presented in bold.

Glucosinolate	Florets	Leaves	Stems
Progoitrin	6.8 ± 1.58 ^c^	9.69 ± 2.02 ^b^	14.83 ± 2.61 ^a^
Sinigrin	10.54 ± 2.75 ^b^	7.48 ± 3.22 ^b^	17.46 ± 6.71 ^a^
Unidentified 1	8.75 ± 2.61 ^b^	7.35 ± 3.13 ^b^	14.06 ± 1.68 ^a^
Unidentified 2	3.56 ± 1.26 ^a^	2.77 ± 1.03 ^a^	3.67 ± 0.45 ^a^
4-Hydroxyglucobrassicin	13.78 ± 3.32 ^b^	23.48 ± 4.65 ^a^	16.84 ± 6.13 ^b^
Glucobrassicin	32.21 ± 3.49 ^b^	59.85 ± 11.96 ^a^	16.09 ± 2.55 ^c^
4-Methoxyglucobrassicin	10.22 ± 2.16 ^c^	51.88 ± 9.27 ^a^	16.61 ± 2.3 ^b^
Neoglucobrassicin	8.17 ± 3.54 ^c^	62.7 ± 13.6 ^a^	25.49 ± 2.96 ^b^
**Total glucosinolates**	**94.02 ± 20.71 ^b^**	**225.2 ± 48.88 ^a^**	**125.05 ± 25.39 ^b^**

Different letters in a row indicate statistically significant (*p*-value < 0.05) differences between the samples.

**Table 3:** Total phenolic, flavonoid and chlorophyll contents in the aerial parts of cauliflower expressed as mean ± SD (*n* = 9).

	Florets	Leaves	Stems
TPC (mg GAE/g DM)	2.76 ± 0.34 ^b^	4.40 ± 0.22 ^a^	2.65 ± 0.52 ^b^
TFC (mg RUT/g DM)	0.29 ± 0.02 ^b^	0.64 ± 0.06 ^a^	0.22 ± 0.02 ^b^
CH_a_ (μg/g DM)	0.38 ± 0.10 ^b^	14.08 ± 0.63 ^a^	0.34 ± 0.09 ^b^
CH_b_ (μg/g DM)	0.66 ± 0.10 ^b^	8.36 ± 1.64 ^a^	0.66 ± 0.10 ^b^
TCH (μg/g DM)	1.04 ± 0.14 ^b^	22.43 ± 2.10 ^a^	1.00 ± 0.13 ^b^

TPC—total phenolic content; TFC—total flavonoid content; CH_a_—chlorophyll a; CH_b_—chlorophyll b; TCH—total chlorophyll. Different letters in a row indicate statistically significant (*p*-value < 0.05) differences between the samples.

**Table 4:** The proximate composition of the aerial parts of cauliflower, expressed in %, as the mean ± SD (*n* = 9).

	Florets	Leaves	Stems
Ash	9.47 ± 0.53 ^b,1^	10.13 ± 0.64 ^b^	11.78 ± 0.74 ^a^
Protein	23.28 ± 1.46 ^a^	20.74 ± 1.68 ^b^	19.12 ± 1.57 ^b^
Fat	1.94 ± 0.24 ^b^	4.68 ± 0.35 ^a^	4.74 ± 0.40 ^a^
Carbohydrates ^2^	65.31 ± 2.31 ^a^	64.45 ± 2.80 ^a^	64.36 ± 2.91 ^a^

^1^ Different letters in a row indicate statistically significant (*p*-value < 0.05) differences between the samples. ^2^ Calculated from the difference.

**Table 5:** The content of individual amino acids in the aerial parts of cauliflower expressed as mean ± SD (*n* = 9) in µmol/g DM. Total content of amino acids is presented in bold.

Amino Acid	Abbrev.	Florets	Leaves	Stems
Essential amino acids				
Valine	VAL	106.54 ± 23.81 ^a^	60.66 ± 11.15 ^b^	100.01 ± 24.52 ^a^
Leucine	LEU	62.7 ± 11.11 ^a^	10.30 ± 2.93 ^c^	23.93 ± 6.2 ^b^
Isoleucine	ILE	64.2 ± 15.64 ^a^	16.43 ± 2.07 ^c^	41.25 ± 7.23 ^b^
Threonine	THR	54.47 ± 6.56 ^b^	75.14 ± 8.74 ^a^	50.4 ± 14.39 ^b^
Phenylalanine	PHE	62.72 ± 15.97 ^a^	18.19 ± 4.2 ^b^	21.99 ± 7.36 ^b^
Lysine	LYS	36.5 ± 7.72 ^a^	7.48 ± 1.35 ^c^	17.51 ± 3.01 ^b^
Histidine	HIS	41.08 ± 13.16 ^a, b^	42.06 ± 10.39 ^a^	30.68 ± 10.92 ^b^
Tryptophan	TRP	15.32 ± 5.66 ^a^	9.13 ± 2.1 ^b^	12.66 ± 5.25 ^a, b^
Non-essential amino acids				
Alanine	ALA	432.07 ± 53.24 ^a^	332.01 ± 46.53 ^b^	341.32 ± 68.92 ^b^
Glycine	GLY	29.54 ± 4.08 ^a^	22.25 ± 5.01 ^b^	33.02 ± 5.51 ^a^
Serine	SER	224.1 ± 32.93 ^b^	276.51 ± 28.78 ^a^	268.31 ± 48.04 ^a^
Proline	PRO	21.27 ± 3.07 ^a^	22.62 ± 4.07 ^a^	21.18 ± 3.99 ^a^
Asparagine	ASN	126.9 ± 14.12 ^c^	444.96 ± 76.12 ^a^	205.97 ± 55.7 ^b^
Glutamic acid	GLU	146.75 ± 24.74 ^b^	93.32 ± 15.21 ^c^	193.56 ± 46.19 ^a^
Glutamine	GLN	491.85 ± 124.21 ^c^	2121.63 ± 314.16 ^a^	1009.42 ± 196.41 ^b^
Tyrosine	TYR	42.86 ± 7.15 ^a^	13.88 ± 3.29 ^c^	26.36 ± 7.8 ^b^
Other amino acids				
α-Aminoadipic acid	AAA	9.72 ± 1.71 ^c^	43.51 ± 7.95 ^a^	25.07 ± 6.25 ^b^
Total essential amino acids		443.53 ± 26.64 ^a^	239.38 ± 26.13 ^c^	298.44 ± 28.22 ^b^
Total non-essential amino acids		1515.34 ± 182.28 ^c^	3327.19 ± 708.33 ^a^	2099.14 ± 324.86 ^b^
**Total amino acid**		**1968.58 ± 364.87 ^b^**	**3610.08 ± 544.06 ^a^**	**2422.64 ± 517.67 ^b^**

Different letters in a row indicate statistically significant (*p*-value < 0.05) differences between the samples.

## Data Availability

Data is contained within the article.
